# *catena*-Poly[[[diaqua­[3-(pyridin-4-yl)benzoato-κ^2^ *O*,*O*′]gadolinium(III)]-bis­[μ-3-(pyridin-4-yl)benzoato-κ^2^ *O*:*O*′]] monohydrate]

**DOI:** 10.1107/S1600536812026153

**Published:** 2012-06-16

**Authors:** Dong-Ying Li, Guo-Ting Li

**Affiliations:** a Department of Environmental and Municipal Engineering, North China University of Water Conservancy and Electric Power, Zhengzhou 450011, People’s Republic of China

## Abstract

In the title coordination polymer, {[Gd(C_12_H_8_NO_2_)_3_(H_2_O)_2_]·H_2_O}_*n*_, the Gd^III^ ion is ligated by one bidentate carboxyl­ate group, four monodentate bridging carboxyl­ate O atoms and two water mol­ecules. The resulting GdO_8_ polyhedron approximates to a square anti­prism. The bridging ligands link the metal ions into a [100] chain, with each pair of adjacent metal ions being bridged by two ligands. Inter-chain O—H⋯O and O—H⋯N hydrogen bonds help to establish the packing.

## Related literature

For metal-organic frameworks containing aromatic carb­oxy­lic ligands, see: Li *et al.* (2010) ▶. For lanthanide metal-organic frameworks based on aromatic carb­oxy­lic ligands, see: Zhang *et al.* (2010 ▶). For transition metal coordination complexes of 3-pyridin-4-yl­benzo­ate, see: Wu *et al.* (2011 ▶).
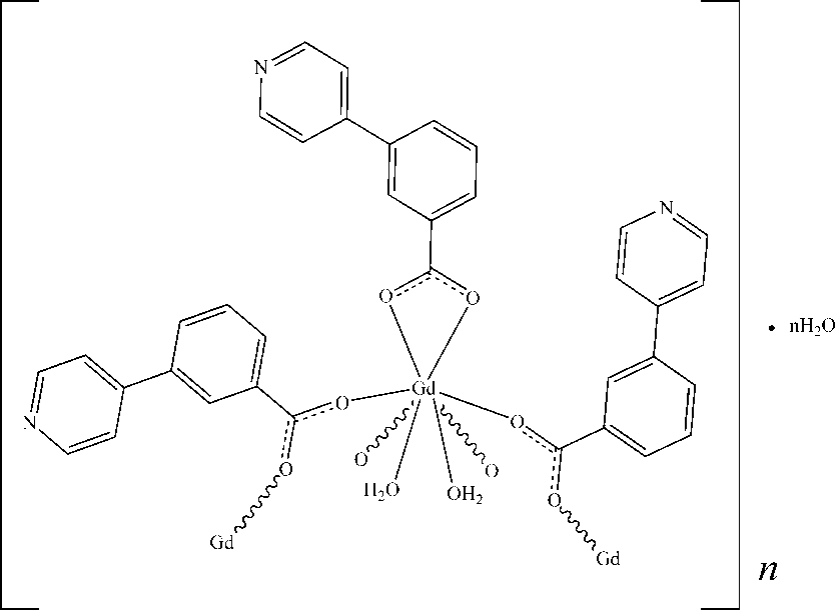


## Experimental

### 

#### Crystal data

[Gd(C_12_H_8_NO_2_)_3_(H_2_O)_2_]·H_2_O
*M* *_r_* = 805.88
Triclinic, 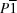

*a* = 9.7252 (5) Å
*b* = 13.9535 (6) Å
*c* = 14.0829 (8) Å
α = 118.019 (5)°
β = 104.240 (5)°
γ = 90.089 (4)°
*V* = 1619.84 (14) Å^3^
*Z* = 2
Mo *K*α radiation
μ = 2.11 mm^−1^
*T* = 293 K
0.35 × 0.21 × 0.18 mm

#### Data collection

Siemens SMART CCD diffractometer
Absorption correction: multi-scan (*SADABS*; Sheldrick, 1996 ▶) *T*_min_ = 0.970, *T*_max_ = 1.000
12338 measured reflections
5702 independent reflections
4844 reflections with *I* > 2σ(*I*)
*R*_int_ = 0.023

#### Refinement

*R*[*F* ^2^ > 2σ(*F* ^2^)] = 0.025
*wR*(*F* ^2^) = 0.061
*S* = 1.05
5702 reflections
460 parameters
2 restraints
H atoms treated by a mixture of independent and constrained refinement
Δρ_max_ = 0.85 e Å^−3^
Δρ_min_ = −0.66 e Å^−3^

### 

Data collection: *SMART* (Siemens, 1996 ▶); cell refinement: *SAINT* (Siemens, 1996 ▶); data reduction: *SAINT*; program(s) used to solve structure: *SHELXS97* (Sheldrick, 2008 ▶); program(s) used to refine structure: *SHELXL97* (Sheldrick, 2008 ▶); molecular graphics: *SHELXTL* (Sheldrick, 2008 ▶); software used to prepare material for publication: *SHELXL97*.

## Supplementary Material

Crystal structure: contains datablock(s) I, global. DOI: 10.1107/S1600536812026153/hb6834sup1.cif

Structure factors: contains datablock(s) I. DOI: 10.1107/S1600536812026153/hb6834Isup2.hkl

Additional supplementary materials:  crystallographic information; 3D view; checkCIF report

## Figures and Tables

**Table 1:** Selected bond lengths (Å)

Gd1—O5^i^	2.290 (2)
Gd1—O4^ii^	2.295 (2)
Gd1—O3	2.395 (2)
Gd1—O6	2.405 (2)
Gd1—O8	2.443 (2)
Gd1—O7	2.447 (2)
Gd1—O2	2.468 (2)
Gd1—O1	2.532 (2)

Symmetry codes: (i) 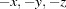; (ii) 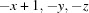.

**Table 2:** Hydrogen-bond geometry (Å, °)

*D*—H⋯*A*	*D*—H	H⋯*A*	*D*⋯*A*	*D*—H⋯*A*
O7—H7*A*⋯O1^i^	0.71 (3)	2.12 (3)	2.826 (3)	173 (4)
O7—H7*B*⋯N1^iii^	0.89 (4)	1.89 (4)	2.772 (4)	173 (3)
O8—H8*B*⋯O2^ii^	0.75 (3)	2.05 (3)	2.793 (3)	173 (4)
O8—H8*A*⋯N3^iv^	0.84 (4)	1.95 (4)	2.789 (4)	175 (3)

Symmetry codes: (i) 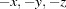; (ii) 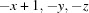; (iii) 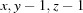; (iv) 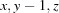.

**Fig. 1.**
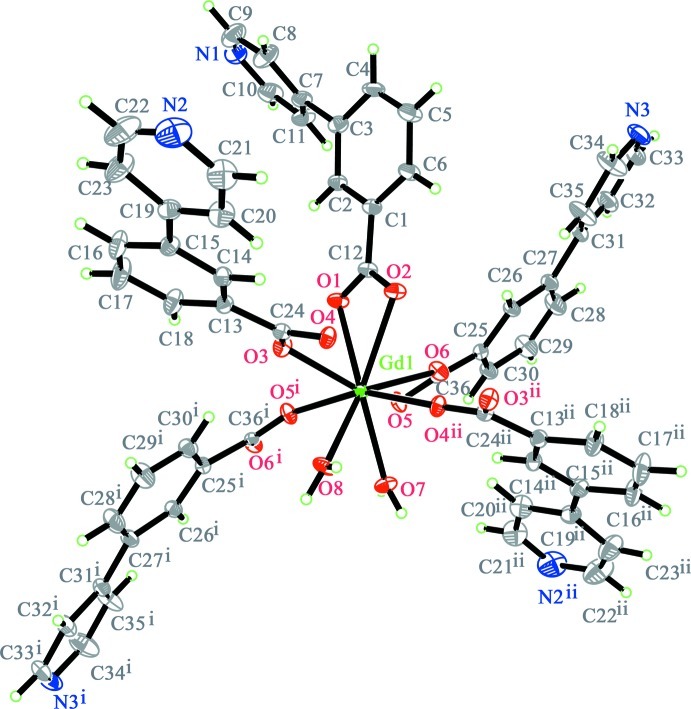
Coordination environment of the Gd atom in (1). Displacement ellipsoids are drawn at the 30% probability level. Symmetry code: (i) -x, -y, -z; (ii) -x + 1, -y, -z.

**Fig. 2.**
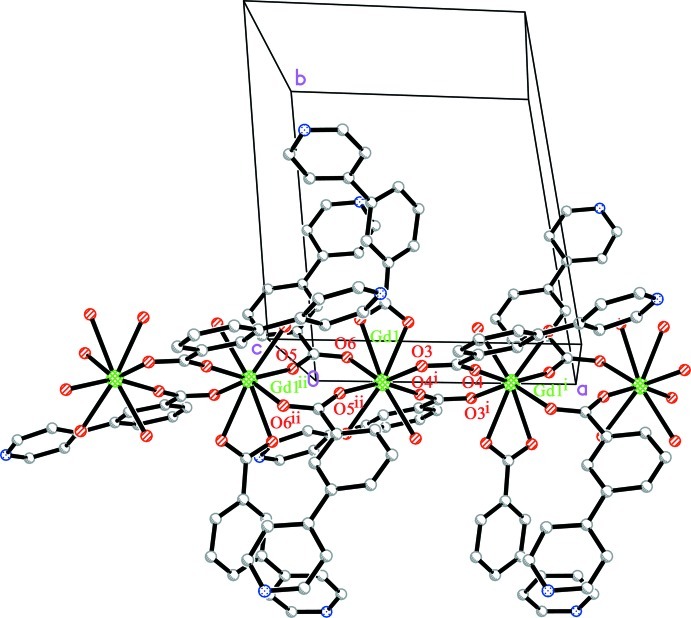
View of infinite two-stranded chain bridged by L in (1). Symmetry code: (i) -x, -y, -z; (ii) -x + 1, -y, -z.

## supplementary crystallographic information

### Comment

Aromatic carboxylic ligands have been widely used to construct metal-organic frameworks (MOFs) with novel structures and unique properties (Li *et al.*, 2010). Especially, lanthanide MOFs of aromatic carboxylic ligands have largely drawn current attention (Zhang, *et al.*, 2010) owing to their potential applications in medical imaging, sensors and electro-optical devices. 3-Pyridin-4-ylbenzoic acid (HL) which possess a pyridyl group and a benzoic acid group is a typical unsymmetrical spacer, and up to now only a serial of its transition metal coordination complexes was synthesized and characterized (Wu, *et al.*, 2011). Herein we report the synthesis and structure of a gadolinium(III) complex of deprotonated 3-pyridin-4-ylbenzoic acid (HL), namely, [Gd(*L*)_3_(H_2_O)_2_]_n_ (1).n(H_2_O).

In (1), the Gd atom is in an eight-coordinate environment of O_8_ ligated by six carboxylato O atoms from five ligands *L* and two O atoms from water molecules (Fig. 1). The Gd—O bonds fall in the normal range from 2.290 (2) to 2.532 (2) Å. In (1), deprotonated ligands *L* act as bidentate ligands, and display two coordinating modes, namely, chelating and bridging coordination models of carboxyl groups without the nitrogen atoms of the pyridyl groups taking part in the coordination. Complex (1) is a two-stranded polymeric chain builted from two *L* simultaneously bridging adjacent two Gd side by side and two *L* chelating them up and down with the separations of the adjacent metal Gd ions bridged by the carboxylato O3 and O4 of one *L*, and by the carboxylato O5 and O6 of the other *L* being 4.833 and 4.895 Å, respectively (Fig. 2). Apart from intrachain O—H···O hydrogen bonds, the chains of complex (1) are connected by interchain O—H···N hydrogen bonds between the coordinated water molecules (as donors) and the uncoordinated pyridyl groups of *L* (as acceptors), leading to the formation of a three-dimensional network structure with uncoordinated water molecules residing in the accessible void.

### Experimental

A mixture of Gd(NO_3_)_3_ (0.045 g, 0.1 mmol), HL (0.060 g, 0.3 mmol), NaOH (0.012 0.3 mmol) and deionized water (10 ml) was sealed into a 25 ml Teflon-lined stainless autoclave. The autoclave was heated at 180 °C for four days. As cooled to room temperature gradually, colourless block crystals of (I) were obtained in 37% yield (based on Gd). Selected IR (cm^-1^, KBr pellet): 3440(*s*), 1611(*m*), 1545(*m*), 1436(*s*), 1396(*s*), 1384(*s*), 1282(*w*), 1173(*s*), 1131(*s*), 766(*m*), 719(*m*), 655(*m*).

### Refinement

The H atoms of water were located from difference Fourier maps and included in the final refinement by using geometrical restrains, while the other hydrogen atom positions were generated geometrically and these H atoms were allowed to ride on their parent atoms.

### Crystal data

**Table:**

[Gd(C_12_H_8_NO_2_)_3_(H_2_O)_2_]·H_2_O	*Z* = 2
*M**_r_* = 805.88	*F*(000) = 806
Triclinic, *P*1	*D*_x_ = 1.652 Mg m^−^^3^
Hall symbol: -P 1	Mo *K*α radiation, λ = 0.71073 Å
*a* = 9.7252 (5) Å	Cell parameters from 8413 reflections
*b* = 13.9535 (6) Å	θ = 2.9–26.3°
*c* = 14.0829 (8) Å	µ = 2.11 mm^−^^1^
α = 118.019 (5)°	*T* = 293 K
β = 104.240 (5)°	Block, colourless
γ = 90.089 (4)°	0.35 × 0.21 × 0.18 mm
*V* = 1619.84 (14) Å^3^	

### Data collection

**Table:**

Siemens SMART CCD diffractometer	5702 independent reflections
Radiation source: fine-focus sealed tube	4844 reflections with *I* > 2σ(*I*)
Graphite monochromator	*R*_int_ = 0.023
ω scan	θ_max_ = 25.0°, θ_min_ = 2.9°
Absorption correction: multi-scan (*SADABS*; Sheldrick, 1996)	*h* = −11→11
*T*_min_ = 0.970, *T*_max_ = 1.000	*k* = −16→16
12338 measured reflections	*l* = −16→15

### Refinement

**Table:**

Refinement on *F*^2^	Primary atom site location: structure-invariant direct methods
Least-squares matrix: full	Secondary atom site location: difference Fourier map
*R*[*F*^2^ > 2σ(*F*^2^)] = 0.025	Hydrogen site location: inferred from neighbouring sites
*wR*(*F*^2^) = 0.061	H atoms treated by a mixture of independent and constrained refinement
*S* = 1.05	*w* = 1/[σ^2^(*F*_o_^2^) + (0.0298*P*)^2^ + 0.5641*P*] where *P* = (*F*_o_^2^ + 2*F*_c_^2^)/3
5702 reflections	(Δ/σ)_max_ = 0.001
460 parameters	Δρ_max_ = 0.85 e Å^−^^3^
2 restraints	Δρ_min_ = −0.66 e Å^−^^3^

### Special details

**Table:**

Geometry. All e.s.d.'s (except the e.s.d. in the dihedral angle between two l.s. planes) are estimated using the full covariance matrix. The cell e.s.d.'s are taken into account individually in the estimation of e.s.d.'s in distances, angles and torsion angles; correlations between e.s.d.'s in cell parameters are only used when they are defined by crystal symmetry. An approximate (isotropic) treatment of cell e.s.d.'s is used for estimating e.s.d.'s involving l.s. planes.
Refinement. Refinement of *F*^2^ against ALL reflections. The weighted *R*-factor *wR* and goodness of fit *S* are based on *F*^2^, conventional *R*-factors *R* are based on *F*, with *F* set to zero for negative *F*^2^. The threshold expression of *F*^2^ > σ(*F*^2^) is used only for calculating *R*-factors(gt) *etc*. and is not relevant to the choice of reflections for refinement. *R*-factors based on *F*^2^ are statistically about twice as large as those based on *F*, and *R*- factors based on ALL data will be even larger.

### Fractional atomic coordinates and isotropic or equivalent isotropic displacement parameters (Å^2^)

**Table:**

	*x*	*y*	*z*	*U*_iso_*/*U*_eq_	
Gd1	0.252518 (15)	−0.002199 (12)	0.002647 (12)	0.01875 (6)	
O1	0.2075 (2)	0.17870 (18)	0.15035 (19)	0.0298 (5)	
O2	0.3835 (2)	0.18197 (17)	0.07980 (19)	0.0290 (5)	
O3	0.4213 (2)	0.03112 (19)	0.17419 (19)	0.0307 (5)	
O4	0.6183 (2)	0.02697 (19)	0.12145 (18)	0.0284 (5)	
O5	−0.0958 (2)	0.04584 (19)	−0.0786 (2)	0.0310 (5)	
O6	0.1155 (2)	0.08726 (18)	−0.0915 (2)	0.0295 (5)	
O7	0.0774 (2)	−0.1512 (2)	−0.1568 (2)	0.0312 (6)	
O8	0.3516 (3)	−0.17299 (19)	−0.0343 (2)	0.0333 (6)	
O9	0.3749 (6)	0.2281 (5)	−0.1164 (5)	0.1291 (19)	
N1	0.1269 (4)	0.6874 (3)	0.6483 (3)	0.0487 (9)	
N2	1.3290 (4)	0.2014 (3)	0.5396 (4)	0.0674 (11)	
N3	0.2287 (4)	0.6204 (3)	−0.0892 (3)	0.0560 (10)	
C1	0.3436 (3)	0.3525 (3)	0.2204 (3)	0.0268 (7)	
C2	0.2774 (3)	0.4068 (3)	0.3048 (3)	0.0285 (7)	
H2	0.2038	0.3674	0.3114	0.034*	
C3	0.3178 (4)	0.5181 (3)	0.3797 (3)	0.0320 (8)	
C4	0.4266 (4)	0.5753 (3)	0.3694 (3)	0.0384 (9)	
H4	0.4578	0.6507	0.4217	0.046*	
C5	0.4884 (4)	0.5218 (3)	0.2832 (3)	0.0441 (10)	
H5	0.5597	0.5613	0.2746	0.053*	
C6	0.4478 (4)	0.4114 (3)	0.2092 (3)	0.0349 (8)	
H6	0.4914	0.3755	0.1503	0.042*	
C7	0.2509 (4)	0.5773 (3)	0.4722 (3)	0.0343 (8)	
C8	0.3328 (5)	0.6354 (3)	0.5820 (3)	0.0514 (11)	
H8	0.4341	0.6393	0.5998	0.062*	
C9	0.2673 (5)	0.6879 (4)	0.6658 (4)	0.0609 (13)	
H9	0.3263	0.7268	0.7408	0.073*	
C10	0.0485 (5)	0.6331 (3)	0.5419 (4)	0.0509 (11)	
H10	−0.0522	0.6332	0.5267	0.061*	
C11	0.1031 (4)	0.5768 (3)	0.4523 (3)	0.0444 (10)	
H11	0.0413	0.5384	0.3782	0.053*	
C12	0.3088 (3)	0.2315 (3)	0.1464 (3)	0.0246 (7)	
C13	0.6422 (3)	0.0531 (3)	0.3047 (3)	0.0264 (7)	
C14	0.7906 (3)	0.0751 (3)	0.3354 (3)	0.0276 (7)	
H14	0.8363	0.0731	0.2823	0.033*	
C15	0.8737 (3)	0.0999 (3)	0.4416 (3)	0.0299 (8)	
C16	0.8041 (4)	0.0999 (4)	0.5168 (3)	0.0477 (11)	
H16	0.8584	0.1169	0.5901	0.057*	
C17	0.6570 (4)	0.0754 (4)	0.4861 (3)	0.0563 (12)	
H17	0.6113	0.0735	0.5377	0.068*	
C18	0.5758 (4)	0.0535 (3)	0.3811 (3)	0.0404 (9)	
H18	0.4744	0.0387	0.3614	0.048*	
C19	1.0316 (4)	0.1321 (3)	0.4756 (3)	0.0341 (8)	
C20	1.1051 (4)	0.1240 (3)	0.3992 (4)	0.0446 (10)	
H20	1.0552	0.0931	0.3220	0.053*	
C21	1.2497 (4)	0.1605 (4)	0.4350 (4)	0.0548 (11)	
H21	1.2952	0.1559	0.3808	0.066*	
C22	1.2615 (5)	0.2065 (4)	0.6113 (4)	0.0720 (15)	
H22	1.3162	0.2343	0.6871	0.086*	
C23	1.1152 (4)	0.1742 (4)	0.5848 (4)	0.0566 (12)	
H23	1.0733	0.1812	0.6416	0.068*	
C24	0.5544 (3)	0.0352 (3)	0.1922 (3)	0.0236 (7)	
C25	−0.0766 (3)	0.1751 (3)	−0.1399 (3)	0.0234 (7)	
C26	0.0122 (3)	0.2600 (3)	−0.1274 (3)	0.0268 (7)	
H26	0.1128	0.2649	−0.0993	0.032*	
C27	−0.0424 (4)	0.3386 (3)	−0.1553 (3)	0.0314 (8)	
C28	−0.1895 (4)	0.3258 (3)	−0.2000 (4)	0.0465 (10)	
H28	−0.2296	0.3774	−0.2209	0.056*	
C29	−0.2787 (4)	0.2405 (3)	−0.2148 (4)	0.0463 (10)	
H29	−0.3789	0.2332	−0.2466	0.056*	
C30	−0.2230 (4)	0.1656 (3)	−0.1837 (3)	0.0316 (8)	
H30	−0.2848	0.1077	−0.1922	0.038*	
C31	0.0522 (4)	0.4346 (3)	−0.1337 (3)	0.0349 (8)	
C32	−0.0024 (5)	0.5242 (3)	−0.1383 (3)	0.0491 (11)	
H32	−0.1030	0.5246	−0.1565	0.059*	
C33	0.0871 (6)	0.6126 (3)	−0.1170 (4)	0.0559 (12)	
H33	0.0448	0.6718	−0.1224	0.067*	
C34	0.2818 (5)	0.5352 (4)	−0.0836 (5)	0.0685 (14)	
H34	0.3830	0.5385	−0.0628	0.082*	
C35	0.2005 (5)	0.4419 (3)	−0.1060 (4)	0.0586 (13)	
H35	0.2459	0.3830	−0.1024	0.070*	
C36	−0.0144 (3)	0.0968 (2)	−0.1011 (2)	0.0203 (7)	
H7A	0.004 (4)	−0.156 (3)	−0.158 (3)	0.030*	
H8A	0.318 (4)	−0.235 (3)	−0.047 (3)	0.030*	
H7B	0.088 (3)	−0.207 (3)	−0.219 (3)	0.030*	
H8B	0.422 (4)	−0.181 (3)	−0.048 (3)	0.030*	
H9B	0.450 (3)	0.198 (3)	−0.132 (3)	0.030*	
H9A	0.359 (4)	0.189 (3)	−0.087 (3)	0.030*	

### Atomic displacement parameters (Å^2^)

**Table:**

	*U*^11^	*U*^22^	*U*^33^	*U*^12^	*U*^13^	*U*^23^
Gd1	0.01859 (9)	0.01705 (9)	0.02183 (9)	0.00224 (6)	0.00914 (6)	0.00871 (7)
O1	0.0271 (12)	0.0209 (12)	0.0364 (14)	0.0000 (10)	0.0167 (11)	0.0063 (11)
O2	0.0239 (12)	0.0224 (12)	0.0353 (14)	0.0021 (10)	0.0154 (11)	0.0065 (11)
O3	0.0215 (12)	0.0426 (15)	0.0293 (13)	0.0027 (10)	0.0080 (10)	0.0181 (12)
O4	0.0270 (12)	0.0393 (14)	0.0239 (12)	0.0062 (10)	0.0126 (10)	0.0164 (11)
O5	0.0294 (13)	0.0361 (14)	0.0416 (15)	0.0049 (11)	0.0149 (11)	0.0274 (13)
O6	0.0198 (12)	0.0311 (14)	0.0434 (15)	0.0055 (10)	0.0073 (11)	0.0234 (12)
O7	0.0210 (13)	0.0284 (14)	0.0306 (14)	0.0001 (11)	0.0099 (12)	0.0023 (11)
O8	0.0273 (14)	0.0206 (13)	0.0557 (17)	0.0068 (11)	0.0211 (13)	0.0170 (13)
O9	0.155 (5)	0.143 (5)	0.166 (5)	0.077 (4)	0.082 (4)	0.117 (4)
N1	0.067 (3)	0.035 (2)	0.044 (2)	0.0145 (18)	0.029 (2)	0.0121 (17)
N2	0.037 (2)	0.074 (3)	0.074 (3)	−0.008 (2)	0.010 (2)	0.025 (2)
N3	0.078 (3)	0.032 (2)	0.068 (3)	−0.0022 (19)	0.033 (2)	0.0253 (19)
C1	0.0235 (17)	0.0210 (18)	0.0311 (19)	0.0030 (14)	0.0040 (15)	0.0107 (15)
C2	0.0273 (18)	0.0241 (18)	0.0302 (19)	0.0036 (14)	0.0093 (15)	0.0096 (16)
C3	0.0324 (19)	0.0254 (19)	0.032 (2)	0.0078 (15)	0.0069 (16)	0.0101 (16)
C4	0.046 (2)	0.0195 (19)	0.037 (2)	−0.0030 (16)	0.0090 (18)	0.0049 (17)
C5	0.047 (2)	0.028 (2)	0.055 (3)	−0.0019 (17)	0.022 (2)	0.014 (2)
C6	0.037 (2)	0.0244 (19)	0.042 (2)	0.0016 (16)	0.0195 (18)	0.0117 (17)
C7	0.043 (2)	0.0203 (18)	0.033 (2)	0.0084 (16)	0.0128 (17)	0.0071 (16)
C8	0.052 (3)	0.050 (3)	0.036 (2)	0.022 (2)	0.012 (2)	0.008 (2)
C9	0.081 (4)	0.050 (3)	0.035 (2)	0.022 (3)	0.013 (2)	0.009 (2)
C10	0.053 (3)	0.036 (2)	0.058 (3)	0.009 (2)	0.029 (2)	0.012 (2)
C11	0.045 (2)	0.034 (2)	0.039 (2)	−0.0008 (18)	0.0153 (19)	0.0045 (18)
C12	0.0183 (16)	0.0211 (17)	0.0310 (19)	0.0031 (13)	0.0070 (14)	0.0098 (15)
C13	0.0256 (18)	0.0285 (19)	0.0255 (18)	0.0029 (14)	0.0093 (15)	0.0124 (15)
C14	0.0267 (18)	0.0315 (19)	0.0283 (19)	0.0052 (15)	0.0116 (15)	0.0156 (16)
C15	0.0251 (18)	0.033 (2)	0.0283 (19)	0.0023 (15)	0.0048 (15)	0.0136 (16)
C16	0.040 (2)	0.077 (3)	0.032 (2)	−0.002 (2)	0.0036 (18)	0.033 (2)
C17	0.039 (2)	0.103 (4)	0.037 (2)	−0.003 (2)	0.0124 (19)	0.041 (3)
C18	0.0252 (19)	0.067 (3)	0.032 (2)	−0.0013 (18)	0.0079 (16)	0.026 (2)
C19	0.0296 (19)	0.033 (2)	0.036 (2)	0.0032 (16)	0.0070 (17)	0.0143 (17)
C20	0.027 (2)	0.055 (3)	0.050 (3)	0.0052 (18)	0.0067 (18)	0.026 (2)
C21	0.034 (2)	0.067 (3)	0.066 (3)	0.006 (2)	0.016 (2)	0.034 (3)
C22	0.040 (3)	0.086 (4)	0.051 (3)	−0.009 (3)	−0.011 (2)	0.014 (3)
C23	0.037 (2)	0.079 (3)	0.037 (2)	−0.004 (2)	0.0046 (19)	0.017 (2)
C24	0.0217 (17)	0.0223 (17)	0.0269 (18)	0.0034 (13)	0.0084 (14)	0.0112 (15)
C25	0.0238 (17)	0.0247 (18)	0.0242 (17)	0.0043 (14)	0.0076 (14)	0.0134 (15)
C26	0.0243 (17)	0.0266 (18)	0.0304 (19)	0.0049 (14)	0.0065 (15)	0.0151 (16)
C27	0.038 (2)	0.0240 (19)	0.036 (2)	0.0067 (15)	0.0121 (17)	0.0170 (17)
C28	0.043 (2)	0.045 (3)	0.064 (3)	0.0128 (19)	0.008 (2)	0.039 (2)
C29	0.025 (2)	0.053 (3)	0.069 (3)	0.0071 (18)	0.0040 (19)	0.041 (2)
C30	0.0281 (19)	0.032 (2)	0.037 (2)	0.0001 (15)	0.0078 (16)	0.0185 (17)
C31	0.050 (2)	0.028 (2)	0.035 (2)	0.0037 (17)	0.0189 (18)	0.0182 (17)
C32	0.060 (3)	0.037 (2)	0.057 (3)	0.003 (2)	0.010 (2)	0.031 (2)
C33	0.095 (4)	0.031 (2)	0.051 (3)	0.007 (2)	0.021 (3)	0.027 (2)
C34	0.059 (3)	0.048 (3)	0.112 (4)	0.001 (2)	0.036 (3)	0.044 (3)
C35	0.055 (3)	0.041 (3)	0.101 (4)	0.010 (2)	0.033 (3)	0.046 (3)
C36	0.0264 (18)	0.0155 (16)	0.0164 (16)	−0.0001 (13)	0.0068 (13)	0.0054 (13)

### Geometric parameters (Å, º)

**Table:**

Gd1—O5^i^	2.290 (2)	C9—H9	0.9500
Gd1—O4^ii^	2.295 (2)	C10—C11	1.375 (5)
Gd1—O3	2.395 (2)	C10—H10	0.9500
Gd1—O6	2.405 (2)	C11—H11	0.9500
Gd1—O8	2.443 (2)	C13—C18	1.382 (4)
Gd1—O7	2.447 (2)	C13—C14	1.389 (4)
Gd1—O2	2.468 (2)	C13—C24	1.505 (4)
Gd1—O1	2.532 (2)	C14—C15	1.388 (4)
O1—C12	1.257 (4)	C14—H14	0.9500
O2—C12	1.270 (4)	C15—C16	1.390 (5)
O3—C24	1.252 (3)	C15—C19	1.491 (5)
O4—C24	1.261 (3)	C16—C17	1.381 (5)
O4—Gd1^ii^	2.295 (2)	C16—H16	0.9500
O5—C36	1.252 (3)	C17—C18	1.379 (5)
O5—Gd1^i^	2.290 (2)	C17—H17	0.9500
O6—C36	1.251 (3)	C18—H18	0.9500
O7—H7A	0.71 (3)	C19—C23	1.380 (5)
O7—H7B	0.89 (4)	C19—C20	1.395 (5)
O8—H8A	0.84 (4)	C20—C21	1.378 (5)
O8—H8B	0.75 (3)	C20—H20	0.9500
O9—H9B	0.872 (10)	C21—H21	0.9500
O9—H9A	0.868 (10)	C22—C23	1.394 (6)
N1—C9	1.325 (5)	C22—H22	0.9500
N1—C10	1.331 (5)	C23—H23	0.9500
N2—C22	1.310 (6)	C25—C26	1.381 (4)
N2—C21	1.320 (6)	C25—C30	1.384 (4)
N3—C33	1.325 (6)	C25—C36	1.502 (4)
N3—C34	1.325 (5)	C26—C27	1.394 (4)
C1—C6	1.387 (4)	C26—H26	0.9500
C1—C2	1.390 (4)	C27—C28	1.387 (5)
C1—C12	1.490 (4)	C27—C31	1.486 (5)
C2—C3	1.391 (5)	C28—C29	1.373 (5)
C2—H2	0.9500	C28—H28	0.9500
C3—C4	1.398 (5)	C29—C30	1.376 (5)
C3—C7	1.488 (5)	C29—H29	0.9500
C4—C5	1.380 (5)	C30—H30	0.9500
C4—H4	0.9500	C31—C32	1.382 (5)
C5—C6	1.379 (5)	C31—C35	1.389 (5)
C5—H5	0.9500	C32—C33	1.374 (6)
C6—H6	0.9500	C32—H32	0.9500
C7—C8	1.377 (5)	C33—H33	0.9500
C7—C11	1.394 (5)	C34—C35	1.381 (5)
C8—C9	1.376 (5)	C34—H34	0.9500
C8—H8	0.9500	C35—H35	0.9500
			
O5^i^—Gd1—O4^ii^	158.30 (9)	O1—C12—C1	120.5 (3)
O5^i^—Gd1—O3	82.32 (8)	O2—C12—C1	119.3 (3)
O4^ii^—Gd1—O3	106.79 (7)	O1—C12—Gd1	61.50 (16)
O5^i^—Gd1—O6	103.62 (7)	O2—C12—Gd1	58.66 (16)
O4^ii^—Gd1—O6	81.10 (7)	C1—C12—Gd1	177.8 (2)
O3—Gd1—O6	143.19 (8)	C18—C13—C14	119.1 (3)
O5^i^—Gd1—O8	89.77 (8)	C18—C13—C24	120.3 (3)
O4^ii^—Gd1—O8	74.67 (8)	C14—C13—C24	120.5 (3)
O3—Gd1—O8	73.33 (9)	C15—C14—C13	121.8 (3)
O6—Gd1—O8	141.74 (9)	C15—C14—H14	119.1
O5^i^—Gd1—O7	74.87 (9)	C13—C14—H14	119.1
O4^ii^—Gd1—O7	85.95 (8)	C14—C15—C16	117.9 (3)
O3—Gd1—O7	138.81 (8)	C14—C15—C19	120.8 (3)
O6—Gd1—O7	76.44 (8)	C16—C15—C19	121.2 (3)
O8—Gd1—O7	72.76 (9)	C17—C16—C15	120.7 (3)
O5^i^—Gd1—O2	125.02 (8)	C17—C16—H16	119.6
O4^ii^—Gd1—O2	76.66 (8)	C15—C16—H16	119.6
O3—Gd1—O2	74.77 (8)	C18—C17—C16	120.5 (4)
O6—Gd1—O2	72.28 (8)	C18—C17—H17	119.7
O8—Gd1—O2	127.98 (8)	C16—C17—H17	119.7
O7—Gd1—O2	146.07 (9)	C17—C18—C13	119.9 (3)
O5^i^—Gd1—O1	74.17 (8)	C17—C18—H18	120.1
O4^ii^—Gd1—O1	126.86 (7)	C13—C18—H18	120.1
O3—Gd1—O1	74.94 (8)	C23—C19—C20	115.1 (3)
O6—Gd1—O1	72.06 (8)	C23—C19—C15	122.8 (3)
O8—Gd1—O1	146.02 (9)	C20—C19—C15	122.1 (3)
O7—Gd1—O1	128.36 (7)	C21—C20—C19	120.4 (4)
O2—Gd1—O1	51.95 (7)	C21—C20—H20	119.8
C12—O1—Gd1	92.62 (18)	C19—C20—H20	119.8
C12—O2—Gd1	95.27 (18)	N2—C21—C20	124.2 (4)
C24—O3—Gd1	126.2 (2)	N2—C21—H21	117.9
C24—O4—Gd1^ii^	174.8 (2)	C20—C21—H21	117.9
C36—O5—Gd1^i^	163.6 (2)	N2—C22—C23	124.8 (4)
C36—O6—Gd1	125.31 (18)	N2—C22—H22	117.6
Gd1—O7—H7A	121 (3)	C23—C22—H22	117.6
Gd1—O7—H7B	131 (2)	C19—C23—C22	119.7 (4)
H7A—O7—H7B	108 (4)	C19—C23—H23	120.2
Gd1—O8—H8A	134 (2)	C22—C23—H23	120.2
Gd1—O8—H8B	120 (3)	O3—C24—O4	123.2 (3)
H8A—O8—H8B	105 (3)	O3—C24—C13	118.2 (3)
H9B—O9—H9A	93 (3)	O4—C24—C13	118.5 (3)
C9—N1—C10	115.9 (4)	C26—C25—C30	119.6 (3)
C22—N2—C21	115.7 (4)	C26—C25—C36	119.8 (3)
C33—N3—C34	115.4 (4)	C30—C25—C36	120.5 (3)
C6—C1—C2	119.2 (3)	C25—C26—C27	121.5 (3)
C6—C1—C12	120.2 (3)	C25—C26—H26	119.3
C2—C1—C12	120.6 (3)	C27—C26—H26	119.3
C1—C2—C3	120.7 (3)	C28—C27—C26	117.2 (3)
C1—C2—H2	119.6	C28—C27—C31	121.4 (3)
C3—C2—H2	119.6	C26—C27—C31	121.4 (3)
C2—C3—C4	119.2 (3)	C29—C28—C27	121.9 (3)
C2—C3—C7	121.6 (3)	C29—C28—H28	119.1
C4—C3—C7	119.2 (3)	C27—C28—H28	119.1
C5—C4—C3	119.8 (3)	C28—C29—C30	120.0 (3)
C5—C4—H4	120.1	C28—C29—H29	120.0
C3—C4—H4	120.1	C30—C29—H29	120.0
C6—C5—C4	120.7 (3)	C29—C30—C25	119.8 (3)
C6—C5—H5	119.7	C29—C30—H30	120.1
C4—C5—H5	119.7	C25—C30—H30	120.1
C5—C6—C1	120.4 (3)	C32—C31—C35	115.2 (3)
C5—C6—H6	119.8	C32—C31—C27	121.8 (3)
C1—C6—H6	119.8	C35—C31—C27	123.0 (3)
C8—C7—C11	116.9 (3)	C33—C32—C31	120.9 (4)
C8—C7—C3	121.2 (3)	C33—C32—H32	119.5
C11—C7—C3	121.8 (3)	C31—C32—H32	119.5
C9—C8—C7	119.7 (4)	N3—C33—C32	124.0 (4)
C9—C8—H8	120.1	N3—C33—H33	118.0
C7—C8—H8	120.1	C32—C33—H33	118.0
N1—C9—C8	124.1 (4)	N3—C34—C35	124.7 (4)
N1—C9—H9	118.0	N3—C34—H34	117.7
C8—C9—H9	118.0	C35—C34—H34	117.7
N1—C10—C11	124.5 (4)	C34—C35—C31	119.7 (4)
N1—C10—H10	117.7	C34—C35—H35	120.1
C11—C10—H10	117.7	C31—C35—H35	120.1
C10—C11—C7	118.8 (4)	O6—C36—O5	123.6 (3)
C10—C11—H11	120.6	O6—C36—C25	118.6 (3)
C7—C11—H11	120.6	O5—C36—C25	117.8 (3)
O1—C12—O2	120.2 (3)		

Symmetry codes: (i) −*x*, −*y*, −*z*; (ii) −*x*+1, −*y*, −*z*.

### Hydrogen-bond geometry (Å, º)

**Table:**

*D*—H···*A*	*D*—H	H···*A*	*D*···*A*	*D*—H···*A*
O7—H7*A*···O1^i^	0.71 (3)	2.12 (3)	2.826 (3)	173 (4)
O7—H7*B*···N1^iii^	0.89 (4)	1.89 (4)	2.772 (4)	173 (3)
O8—H8*B*···O2^ii^	0.75 (3)	2.05 (3)	2.793 (3)	173 (4)
O8—H8*A*···N3^iv^	0.84 (4)	1.95 (4)	2.789 (4)	175 (3)

Symmetry codes: (i) −*x*, −*y*, −*z*; (ii) −*x*+1, −*y*, −*z*; (iii) *x*, *y*−1, *z*−1; (iv) *x*, *y*−1, *z*.

## References

[bb1] Li, X., Wu, B., Wang, R., Zhang, H., Niu, C., Niu, Y. & Hou, H. (2010). *Inorg. Chem.* **49**, 2600–2613. 10.1021/ic901113p 20141153

[bb2] Sheldrick, G. M. (1996). *SADABS* University of Göttingen, Germany.

[bb3] Sheldrick, G. M. (2008). *Acta Cryst.* A**64**, 112–122. 10.1107/S0108767307043930 18156677

[bb4] Siemens (1996). *SAINT* and *SMART* Siemens Analytical X-ray Instruments Inc., Madison, Wisconsin, USA.

[bb5] Wu, B. L., Wang, R. Y., Zhang, H. Y. & Hou, H. W. (2011). *Inorg. Chim. Acta*, **375**, 2–10.

[bb6] Zhang, L. J., Xu, D. H., Zhou, Y. S. & Jiang, F. (2010). *New J. Chem.* **34**, 2470–2478.

